# *Cudrania tricuspidata* Stem Extract Induces Apoptosis via the Extrinsic Pathway in SiHa Cervical Cancer Cells

**DOI:** 10.1371/journal.pone.0150235

**Published:** 2016-03-09

**Authors:** Sae-Bom Kwon, Min-Je Kim, Jin Mo Yang, Hee-Pom Lee, Jin Tae Hong, Heon-Sang Jeong, Eun Suk Kim, Do-Young Yoon

**Affiliations:** 1 Department of Bioscience and Biotechnology, Bio/Molecular Informatics Center, Konkuk University, Seoul, Korea; 2 Department of Chemistry, University of Minnesota, Twin Cities, Minneapolis, MN, 55455, United States of America; 3 College of Pharmacy, Medical Research Center, Chungbuk National University, Osong, Chungbuk, Korea; 4 Agriculture, Life and Environments Sciences, Chungbuk National University, Cheongju, Chungbuk, Korea; 5 Chungcheongbukdo Bio CS, Osong, Chungbuk, Korea; Institute of Biochemistry and Biotechnology, TAIWAN

## Abstract

The focus of this study is the anti-cancer effects of *Cudrania tricuspidata* stem (CTS) extract on cervical cancer cells. The effect of CTS on cell viability was investigated in HPV-positive cervical cancer cells and HaCaT human normal keratinocytes. CTS showed significant dose-dependent cytotoxic effects in cervical cancer cells. However, there was no cytotoxic effect of CTS on HaCaT keratinocytes at concentrations of 0.125–0.5 mg/mL. Based on this cytotoxic effect, we demonstrated that CTS induced apoptosis by down-regulating the E6 and E7 viral oncogenes. Apoptosis was detected by DAPI staining, annexin V-FITC/PI staining, cell cycle analysis, western blotting, RT-PCR, and JC-1 staining in SiHa cervical cancer cells. The mRNA expression levels of extrinsic pathway molecules such as Fas, death receptor 5 (DR5), and TNF-related apoptosis-inducing ligand (TRAIL) were increased by CTS. Furthermore, CTS treatment activated caspase-3/caspase-8 and cleavage of poly (ADP-ribose) polymerase (PARP). However, the mitochondrial membrane potential and expression levels of intrinsic pathway molecules such as Bcl-2, Bcl-xL, Bax, and cytochrome C were not modulated by CTS. Taken together, these results indicate that CTS induced apoptosis by activating the extrinsic pathway, but not the intrinsic pathway, in SiHa cervical cancer cells. These results suggest that CTS can be used as a modulating agent in cervical cancer.

## Introduction

Cervical cancer is one of the most common diseases affecting women worldwide and remains a high cause of mortality among women in developing countries [1–2]. Epidemiological and clinical data suggest that infection with high-risk human papilloma virus (HPV) types, such as types 16 and 18, plays a major role in the multi-factorial etiology of cervical cancer [3]. High-risk HPV oncoproteins E6 and E7 play important roles in maintaining cervical cancer cell growth. Oncoproteins E6 and E7 inactivate tumor suppressor proteins p53 and pRb, respectively [4]. High-risk HPV oncoprotein E6 associates with and degrades p53, while HPV protein E7 competes with E2F for retinoblastoma protein (pRb) binding sites [5].

*Cudrania tricuspidata* is a deciduous tree belonging to the family *Moraceae* that is mainly distributed in Korea, China, and Japan. The entire *C*. *tricuspidata* plant has been exploited as an important folk remedy for cancer in Korea during the last few decades, while it has also been used as a traditional medicine for curing neuritis and inflammation in other parts of Asia [6]. In addition, several effects of *C*. *tricuspidata* extract have been reported, including antioxidant activity [7] and inhibitory effects on nitric oxide synthase [8]. However, the anti-cancer effects of the extract of the stem of *C*. *tricuspidata* on cervical cancer cells have not been investigated.

Thus, the aims of this study were to investigate the anti-cancer activity of *Cudrania tricuspidata* stem (CTS) extract on HPV-positive cervical cancer cells and to investigate the apoptotic mechanisms of CTS. Here, we report that CTS treatment causes apoptosis via the extrinsic pathway, as well as through repression of HPV-16 oncoproteins E6 and E7 and alteration of protein levels of p53 and p-pRb.

## Materials and Methods

### Reagents and antibodies

CellTiter 96 AQ_ueous_ One Solution Cell Proliferation Assay Reagent [MTS, 3-(4, 5-dimethylthiazol-2-yl)-5-(3-carboxymethoxyphenyl)-2-(4-sulfophenyl)-2H-tetrazolium] was purchased from Promega (Madison, WI, USA). Propidium iodide (PI) and 4′,6-diamidino-2-phenylindole (DAPI) stain were purchased from Sigma-Aldrich (St. Louis, MO, USA). NE-PER Nuclear and Cytoplasmic Extraction Reagents were purchased from Pierce (Rockford, IL, USA). Antibodies specific to PARP, caspase-3, caspase-8, p53, Bcl-2, Bcl-xL, Bax, Bid, pRb, p-pRb, and cytochrome C were purchased from Cell Signaling Technology (Beverly, MA, USA). The anti-rabbit IgG horseradish peroxidase (HRP)-conjugated secondary antibody and anti-mouse IgG HRP-conjugated secondary antibody were purchased from Millipore (Billerica, MA, USA). Antibodies specific to p27, p21, and glyceraldehyde 3-phospahte dehydrogenase (GAPDH) were purchased from Santa Cruz Biotechnology (Santa Cruz, CA, USA). JC-1 (5,5′,6,6′-tetrachloro-1,1′,3,3′-tetraethyl benzimidazolycarbocyanine chloride) was purchased from Enzo (Farmingdale, NY, USA). General-caspase inhibitor Z-VAD-fmk and caspase-8 inhibitor Z-IETD-fmk were purchased from R&D systems (Minneapolis, MN, USA). The FITC-Annexin V Apoptosis Detection Kit I was purchased from BD Biosciences (San Jose, CA, USA).

### Methods of extraction

*Cudrania tricuspidata* stem (CTS) extract was purchased from Korea Plant Extract bank (KPEB), Korea Research Institute of Bioscience and Biotechnology (KRIBB) (Ochang, Chungbuk, Korea). In brief, the dried stem of *C*. *tricuspidata* was washed with sterile water and subjected to extraction with methanol (MeOH) at 30°C for 3 days. The solvent was evaporated under reduced pressure to yield a crude extract, as described in a previous report [9].

### High performance liquid chromatography (HPLC) analysis

The extract was dissolved in methanol (HPLC grade) and filtered through a 0.45-μm syringe filter (Millipore, Billerica, MA, USA) prior to HPLC (ACME 9000 system, Younglin, Anyang, Korea) analysis. The mobile phases were 0.1% (v/v) acetic acid in water (A) and 0.1% (v/v) acetic acid acetonitrile (B). The solvent gradient system was as follows: 92:8 (%, v/v) A:B for 2 min, 90:10 (%, v/v) A:B for 27 min, 70:30 (%, v/v) A:B for 50 min, decreased to 10% A at 51 min, 0:100 (%, v/v) A:B for 60 min, and finally 92:8 (%, v/v) A:B at 70 min. The flow rate was 1 mL/min. The injection volume was 20 μL. The UV detector was operated at 280 nm. The separation was performed on an ODS column (5 μm, 4.6 × 250 mm, Agilent Technologies, Santa Clara, CA, USA).

### Cell culture

HPV-16-positive SiHa and CaSki cervical cancer cells were purchased from the American Type Culture Collection (ATCC; Manassas, VA, USA). Cells were cultured in Dulbecco’s modified Eagle’s medium (DMEM; Hyclone Laboratories, Logan, UT, USA) containing 10% (v/v) heat-inactivated fetal bovine serum (FBS; Hyclone Laboratories). Cells were incubated at 37°C in an atmosphere of 5% CO_2_/95% air with saturated humidity.

### Cell viability assays

Cell viability was assessed by the MTS dye reduction assay, which measures mitochondrial respiratory function. Cervical cancer cells were seeded (12 × 10^4^ cells/mL) in 100 μL medium/well in 96-well plates, incubated overnight, and treated with various concentrations of CTS, as mentioned in the figure legends, for 24 h. Cell viability was calculated by assessing MTS metabolism as previously reported [10]. In brief, media samples (100 μL) were removed and incubated with 100 μL of MTS-PMS mix solution for 1 h at 37°C. Optical absorbance was measured at 492 nm using an ELISA reader (Apollo LB 9110, Berthold Technologies GmbH & Co. KG, Bad Wilbad, Germany).

### DAPI staining

Apoptotic nuclear morphology was observed using DAPI staining. SiHa cells were seeded in 2-well slides and treated with the specified concentrations of CTS for 24 h, after which the 2-well slides were washed with phosphate-buffered saline (PBS). Next, SiHa cells were fixed with 4% paraformaldehyde and stained with DAPI staining solution. The 2-well slides were washed with PBS and mounted on microscope slides with mounting solution. Stained cells were observed using fluorescence microscopy (Olympus, Tokyo, Japan).

### Annexin V and propidium iodide staining

Cervical cancer cells (2.5 × 10^5^ cells/mL) were seeded in 60-mm culture dishes and incubated overnight. Cells were treated with CTS for 24 h, harvested using trypsin-EDTA, and washed with PBS. Annexin V and PI staining were performed using the FITC-Annexin V Apoptosis Detection Kit I (BD Biosciences, San Jose, CA, USA) according to the manufacturer’s instructions. Data was analyzed by flow cytometry using a FACSCalibur instrument and CellQuest software (BD Biosciences, San Jose, CA, USA).

### Cell cycle analyses by flow cytometry

The cell cycle was analyzed by propidium iodide (PI) staining and flow cytometry. SiHa cells (2.5 × 10^5^ cells/well) were seeded in 60-mm culture dishes and treated with various concentrations of CTS for 24 h. Cells were harvested with trypsin-EDTA and fixed with 80% ethanol. Next, the cells were washed twice with cold PBS and centrifuged, after which the resulting supernatants were discarded. The pellet was resuspended and stained with PBS containing 50 μg/mL PI and 100 μg/mL RNase A for 20 min in the dark. DNA content was analyzed by flow cytometry using a FACSCalibur instrument and CellQuest software (BD Biosciences, San Jose, CA, USA).

### Real-time quantitative polymerase chain reaction

Cells treated with CTS were harvested. RNA was extracted using an easy-BLUE^TM^ Total RNA Extraction Kit (iNtRon Biotechnology, SungNam, Korea) according to the manufacturer’s instructions as previously reported [10]. cDNA products were obtained using M-MuLV reverse transcriptase (New England Biolabs, Beverly, MA, USA). Real-time quantitative PCR was performed with a relative quantification protocol using Roter-Gene 6000 series software 1.7 (QIAGEN, Venlo, Netherlands) and the SensiFAST^TM^ SYBR NO-ROX Kit (BIOLINE, London, UK). The expression levels of all target genes were normalized to that of housekeeping gene GAPDH. Each sample was run with the following primer sets: E6, 5′-GCA GCC CTT GAA TTA CCC AT-3′ (forward), 5′-CAG AGG TTG GAC AGG GAA GAA-3′ (reverse); E7, 5′-TGA AGG ACA TGG CTT AGA AGT G-3′ (forward), 5′-GGT GCA AGG GTC ACA GTG TT-3′ (reverse); TRAIL, 5′-AAG TTT GTC GTC GTC GGG GT-3′ (forward), 5′-TGG TGC AGG GAC TTC TCT CT-3′ (reverse); Fas, 5′- TGA AGG ACA TGG CTT AGA AGT- 3′ (forward), 5′-GGT GCA AGG GTC ACA GTG TT-3′ (reverse); DR5, 5′-CAG AGG GAT GGT CAA GGT CG- 3′, 5′-TGA TGA TGC CTG ATT CTT TGT GG-3′; and GAPDH, 5′-TGG GCT ACA CTG AGC ACC AG-3′ (forward), 5′-GGG TGT CGT TGT TGA AGT CA-3′ (reverse).

### Western blot analysis

Cells were treated with the specified concentrations of CTS for 24 h, harvested, washed with PBS, and recentrifuged (1,890 × *g*, 5 min, 4°C). The resulting cell pellets were resuspended in lysis buffer containing 50 mM Tris (pH 7.4), 1.5 M sodium chloride, 1 mM EDTA, 1% NP-40, 0.25% sodium deoxycholate, 0.1% sodium dodecyl sulfate (SDS), and a protease inhibitor cocktail. The cell lysates were incubated on ice for 1 h and clarified by centrifugation at 17,010 × *g* for 30 min at 4°C. Protein content was quantified using a Bradford assay (Bio-Rad, Hercules, CA, USA) and a UV spectrophotometer. The cell lysates were separated by 10–12% SDS polyacrylamide gel electrophoresis (SDS-PAGE). Proteins were transferred to polyvinylidene difluoride membranes (PVDF; Millipore, Billerica, MA, USA), which were blocked in 5% non-fat dried milk dissolved in Tris buffered saline containing Tween-20 (2.7 M NaCl, 53.65 mM KCl, 1 M Tris-HCl, pH 7.4, 0.1% Tween-20) for 1 h at room temperature. The membranes were incubated overnight at 4°C with specific primary antibodies. After washing, the membranes were incubated with the secondary antibodies (HRP conjugated anti-rabbit or anti-mouse IgG) for 1 h at room temperature. After washing, the blots were analyzed using West-Zol Plus and a western blot detection system (iNtRON Biotechnology, SungNam, South Korea).

### Nuclear and cytoplasmic fractionation

The CTS-treated cells were collected and fractionated using NE-PER Nuclear and Cytoplasmic Extraction Reagents (Thermo Fisher Scientific Inc., Rockford, IL, USA) according to the manufacturer’s protocol.

### Analysis of mitochondrial membrane potential (MMP)

MMP (∆ψ_m_) was evaluated by JC-1 staining and flow cytometry. SiHa cells were seeded in 60-mm culture dishes (2.5 × 10^5^ cells/well) and treated with various concentrations of CTS. Cells were harvested with trypsin-EDTA and transferred to 1.5-mL tubes. JC-1 (5 μg/mL) was added to the cells and mixed until it was completely dissolved, after which the cells were incubated in the dark for 10 min at 37°C in an incubator. The cells were centrifuged (300 × *g*, 5 min, 4°C), washed twice with PBS, and resuspended in 200 μL PBS. The solutions were divided using a FACSCalibur instrument and analyzed by CellQuest software (BD Biosciences, San Jose, CA, USA). The entire protocol was performed in minimal light.

### Silencing of endogenous HPV16 E6 and E7 expressions by siRNAs

The siRNAs of E6 and E7 and scrambled siRNA were purchased from Dharmacon (Dharmacon, Lafayette,CO). The E6 siRNA sequence and the E7 siRNA sequence were used as described in previously reported [11]. To suppress transcription of the endogenous HPV16 E6 and E7 genes, SiHa cells were transiently co-transfected with the synthetic siRNAs for HPV16 E6 and E7 or a nontargeting siRNA using Lipofectamine RNAiMAX reagent (Invitrogen) according to the manufacturer's instructions.

### Statistical analysis

Data are presented as the mean ± SEM from at least three independent experiments. Statistical significance was assessed with Student’s t-test. *p < 0.05 or **p < 0.005 was considered statistically significant.

## Results

### Identification of phenolic compounds in CTS

**Fig 1:**
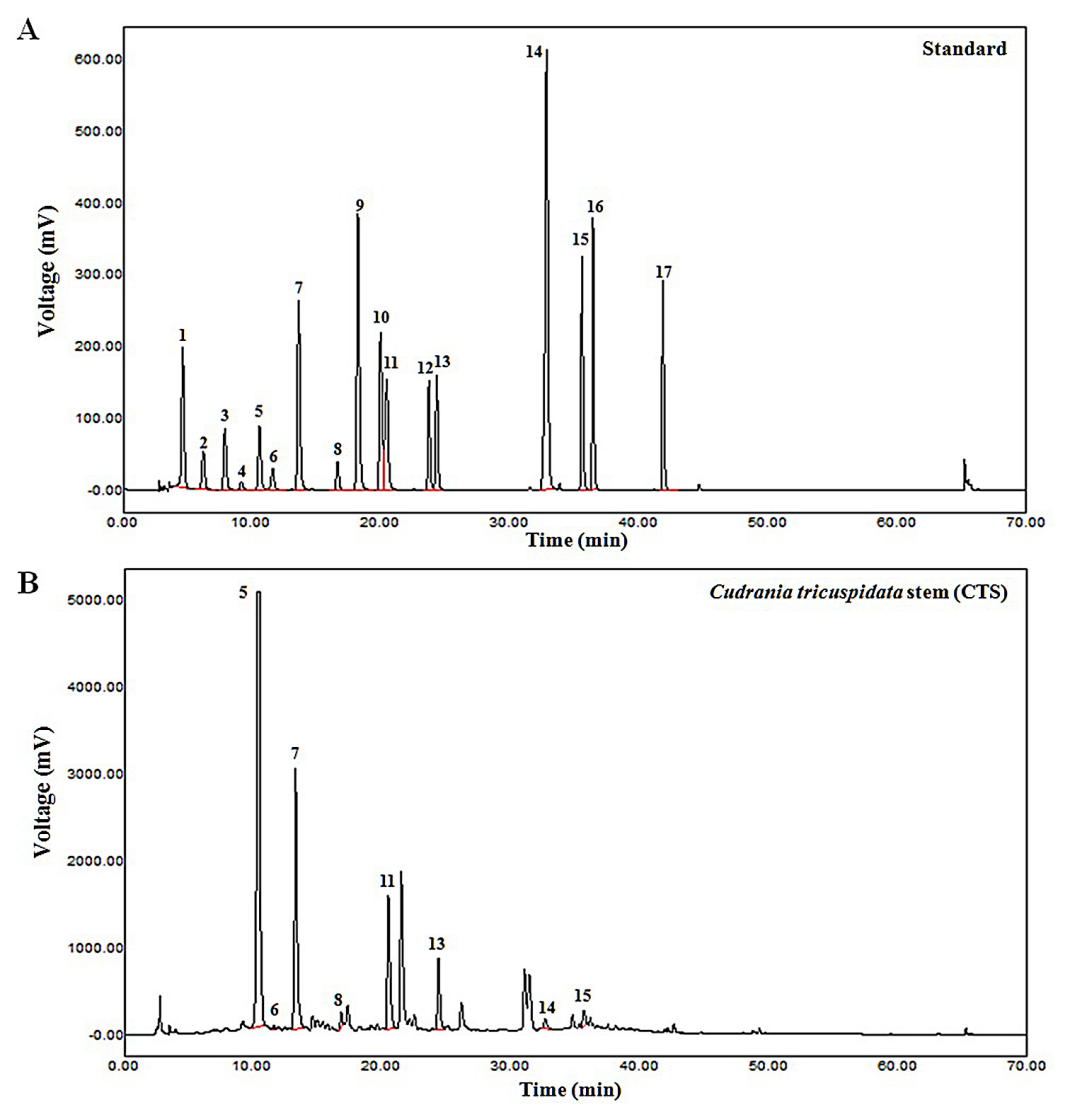
The HPLC analyses of composition in *Cudrania tricuspidata* stem (CTS) extract. The seventeen kinds of the phenolic acid composition of the sample were analyzed by comparing the spectrum of the sample and standards components matched to create a standard curve from Peak area per component to quantify the amount of change. (A) The seventeen kinds of the reference phenolic acid compounds. (B) *Cudrania tricuspidata* stem (CTS) extract. The HPLC analysis showed the presence of eight compounds corresponding to 8 among 17 standard compounds. The 5, 7, 11 peaks represented chlorogenic acid, caffeic acid, and veratric acid, respectively.

We identified potential medicinal components (Fig 1, Table 1) and a large number of chlorogenic acids (Table 2) in the CTS extract using HPLC. Table 2 lists the components in the CTS extract, which included chlorogenic acid, (+)-catechin, caffeic acid, phloretic acid, veratric acid, hesperidin, quercetin, and naringenin. The CTS extract contained diverse phenolic acids and were rich in chlorogenic acid (64.42 mg/g). Chlorogenic acid has been reported to have anticancer and antioxidant properties [12–15]. Quercetin, hesperidin, and other phenolic acids such as caffeic acid have also been reported to exhibit anti-cancer effects in several types of cancer [16–18]. However, these compounds were present at low concentrations in the CTS extract.

**Table 1:** Volatile compounds identified in *Cudrania tricuspidata* stem (CTS) extract.

No.	Phenolic compounds	Standard Curve	CTS extract
1	Gallic acid	y = 30118x + 42.036	Not Detected
2	Homogentisic acid	y = 13708x–2.8616	Not Detected
3	Protocatechuic acid	y = 27223x + 40.724	Not Detected
4	Gentisic acid	y = 3285.2x–4.6768	Not Detected
5	Chlorogenic acid	y = 28445x + 118.08	
6	(+)-Catechin	y = 10473x–62.966	
7	Caffeic acid	y = 60848x + 51.855	
8	Phloretic acid	y = 10075x + 6.1534	
9	ρ-Coumaric acid	y = 94434x + 127.03	Not Detected
10	Ferulic acid	y = 56951x + 62.199	Not Detected
11	Veratric acid	y = 31591x + 15.576	
12	Naringin	y = 32969x + 35.837	Not Detected
13	Hesperidin	y = 28248x + 11.964	
14	Quercetin	y = 25080x–42.462	
15	Naringenin	y = 56671x + 62.852	
16	Hesperitin	y = 61525x + 64.162	Not Detected
17	Biochanin	y = 50770x + 51.274	Not Detected

The seventeen kinds of the reference phenolic acid composition of the sample were analyzed by comparing the spectrum of the sample and standards components matched to create a standard curve from Peak area per component to quantify the amount of change.

**Table 2:** Quantitative HPLC analyses of phenolic compounds in the *Cudrania tricuspidata* stem (CTS) extract.

Phenolic	Peak area			Contents of phenolic compound (mg/g)				
compounds	#1	#2	#3	#1	#2	#3	Average	SD
Chlorogenic acid	89,062.62	93,515.91	92,651.37	62.54	65.67	65.06	64.42	1.66
(+)-Catechin	484.22	431.78	424.37	1.04	0.94	0.93	0.97	0.06
Caffeic acid	37,200.83	37,207.60	36,255.37	12.21	12.21	11.90	12.11	0.18
Phloretic acid	1,378.75	1,559.26	1,425.37	2.72	3.08	2.82	2.88	0.19
Veratric acid	22,186.75	22,509.57	22,365.37	14.04	14.24	14.15	14.14	0.10
Hesperidin	10,541.11	10,737.77	10,657.37	7.45	7.59	7.54	7.53	0.07
Quercetin	1,751.90	1,832.04	1,789.37	1.43	1.49	1.46	1.46	0.03
Naringenin	2,255.98	2,493.72	2,565.37	0.77	0.86	0.88	0.84	0.06
Total				102.21	106.10	104.74	104.35	1.97

The seventeen kinds of the phenolic acid composition of the sample were analyzed by comparing the spectrum of the sample and standards components matched to create a standard curve from Peak area per component to quantify the amount of change. Quantitative HPLC analyses of phenolic compounds were performed three times.

### CTS induces cytotoxic effects in cervical cancer cells and normal keratinocytes

**Fig 2:**
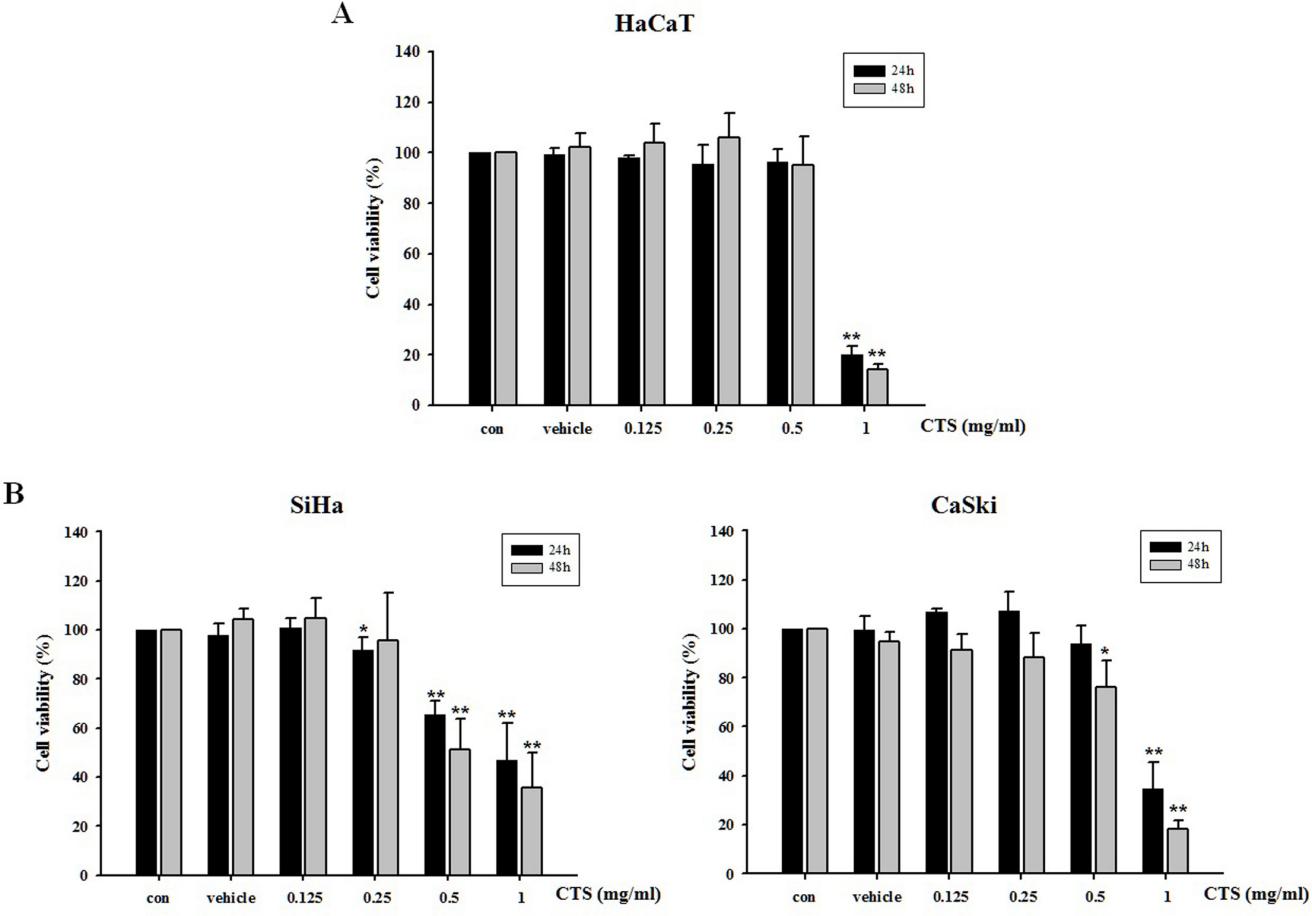
Cytotoxic effects of CTS extract on cervical cancer cells and normal keratinocytes. (A) HaCaT, (B) SiHa and CaSki cells were treated for 24–48 h with various concentrations of CTS extract, after which cell viability was investigated using the MTS assay. Results of *p < 0.05 and **p < 0.005 were considered statistically significant. The CTS-treated cells were compared to the control cells.

The cytotoxic effects of CTS were assessed in several cell lines using the MTS assay. Cervical cancer cell lines and HaCaT normal keratinocytes were treated with various concentrations and time periods (Fig 2). As shown in Fig 2B, the viability of cervical cancer cells was decreased in a dose- and time-dependent manner by CTS extract. The viability of the CaSki HPV16-positive cells was decreased in a time- and dose-dependent manner by CTS extract, but to a lesser degree than that observed in the SiHa HPV16-positive cells. In addition, CTS had no cytotoxic effect in HaCaT human normal keratinocytes concentrations of 0.125–0.5 mg/mL (Fig 2A). Therefore, we decided to perform a study on the mechanism underlying apoptosis induced by CTS extract in SiHa cervical cancer cells.

### CTS induces morphological changes and apoptosis in SiHa cells

**Fig 3:**
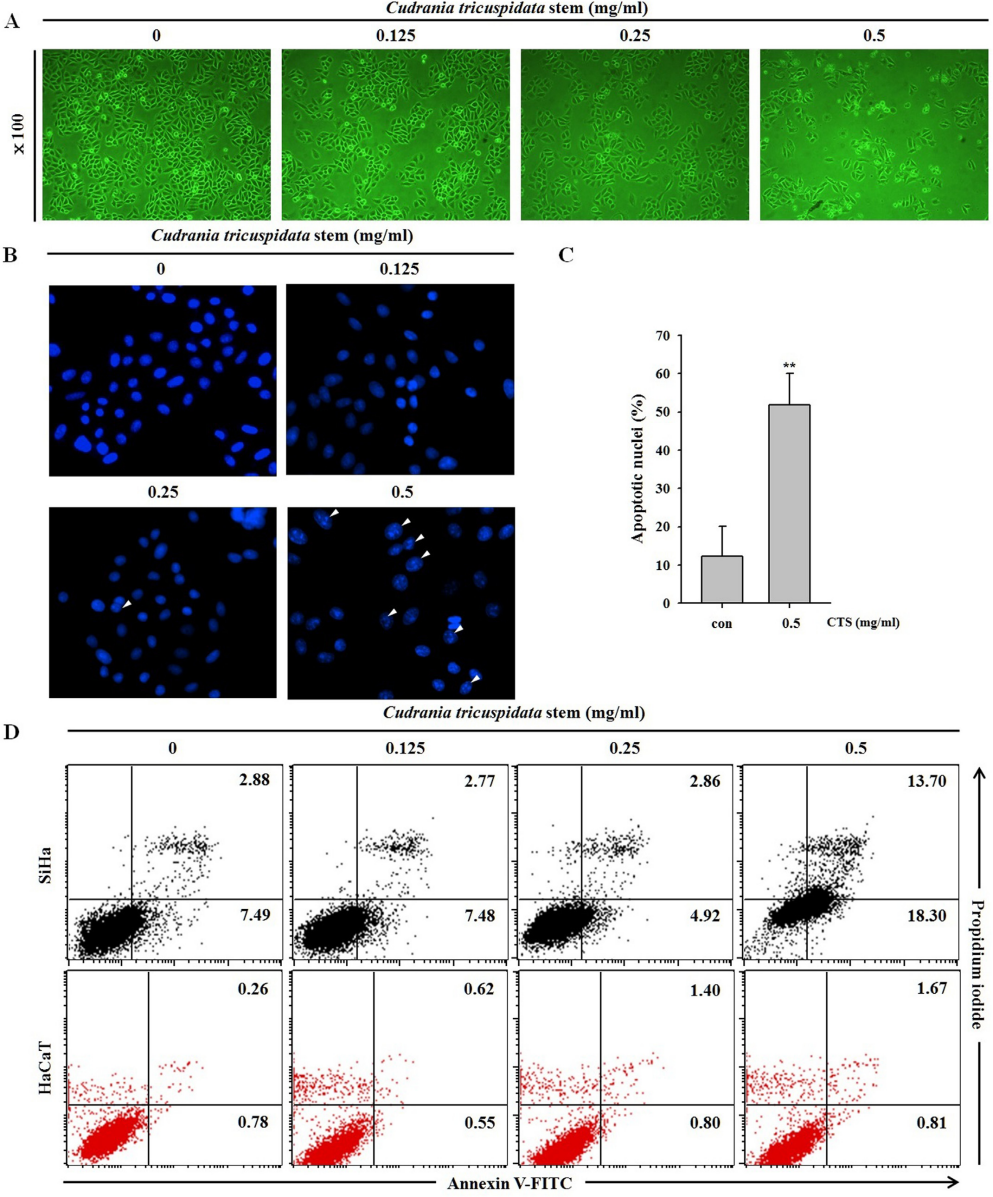
Effects of CTS on SiHa cervical cancer cell morphological changes and apoptosis. (A) Microscopic images of SiHa cells treated with CTS for 24 h. The photographs were taken by phase-contrast microscopy at 100× magnification. (B) Fluorescence microscopic images of SiHa cells treated with CTS for 24 h. Nuclear condensation and chromatin shrinkage were observed. (C) The data on apoptotic nuclei of whole DAPI stained cells were summarized as bar graphs. Results of *p < 0.05 and **p < 0.005 were considered statistically significant. (D) After treatment with the indicated concentration of CTS for 24 h, SiHa and HaCaT cells were stained with annexin V-FITC/PI.

Phase-contrast microscopy showed that CTS induced cell death and morphological changes in SiHa cells in a dose-dependent manner after a 24 h treatment (Fig 3A). DAPI staining was used to observe nuclear condensation, a marker of apoptosis. Nuclear condensation was significantly and dose-dependently increased in the CTS-treated cells in comparison with that of the control cells (Fig 3B and 3C). Annexin V-FITC/PI staining is generally used to detect apoptosis and necrosis. Apoptosis was further confirmed by annexin V-FITC and PI-staining after a 24 h treatment with CTS. The SiHa cells treated with CTS at concentrations of 0.125–0.5 mg/mL for 24 h showed a significantly increased proportion of apoptotic cells in comparison with that of the control cells, indicating that CTS induced apoptosis. However, there were no alterations in CTS treated HaCaT cells (Fig 3D).

### CTS inhibits E6/E7 expression and regulates expression of E6/E7-targeting anti-tumor factors

**Fig 4:**
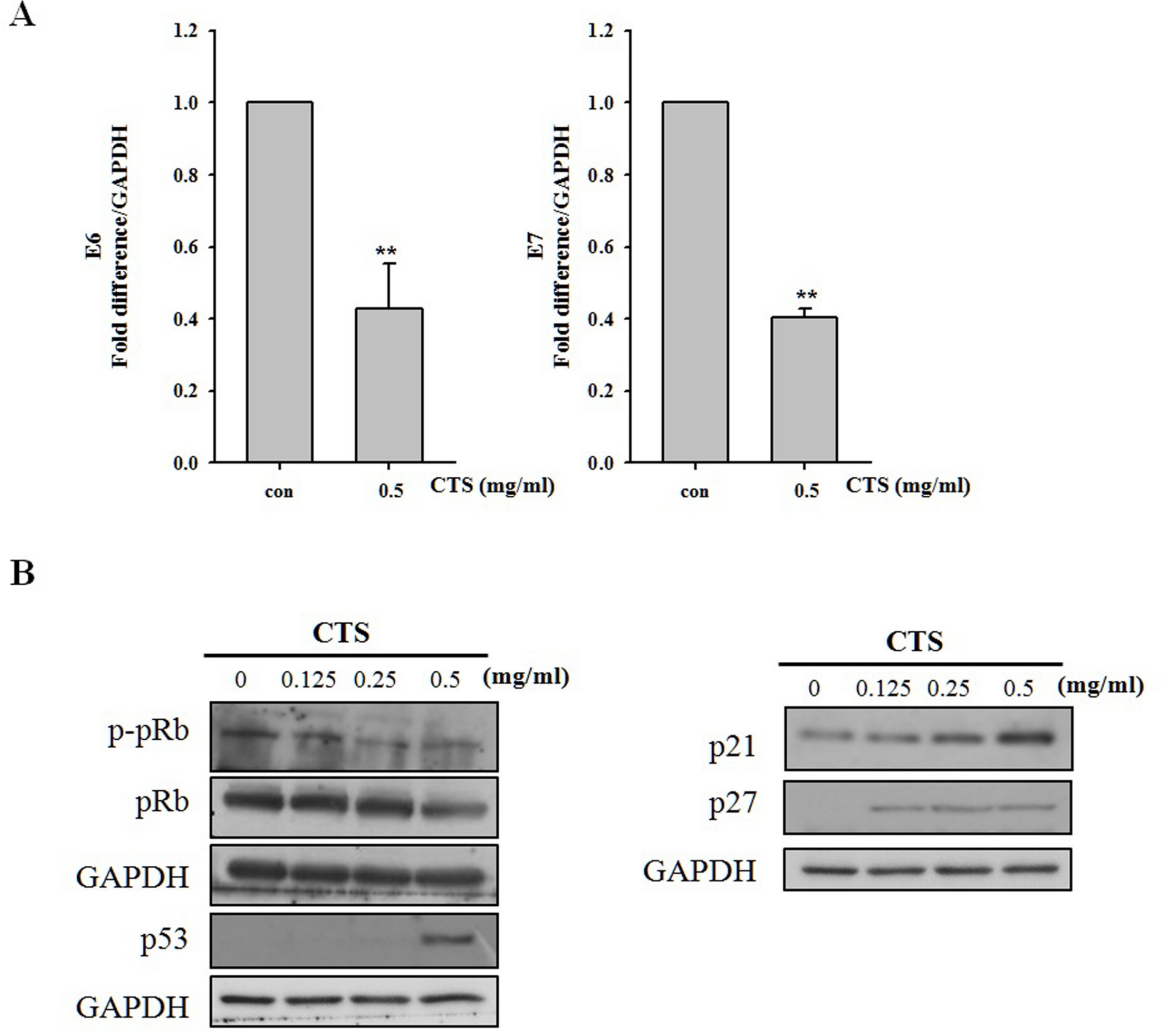
Effects of CTS treatment on oncoprotein E6/E7 mRNA levels and protein levels of E6/E7 targeting p53, pRb, and p-pRb. (A) mRNA levels of oncoproteins E6 and E7 as detected by qRT-PCR. (B) Western blot analyses of pRb, p-pRb, p53, p21, and p27. SiHa cells were treated with the indicated concentration of CTS for 24 h.

E6 and E7 oncoproteins are known to cause degradation of p53 and pRb, respectively. Therefore, down-regulation of E6 and E7 oncogenes would be expected to result in restoration of p53 and pRb levels [19–20]. HPV-16 E6 and E7 mRNA expression levels were investigated by quantitative RT-PCR. E6 mRNA expression was decreased in CTS treated SiHa cells compared with that of the non-treated control cells. In addition, E7 mRNA expression was also decreased (Fig 4A). The expression level of p-pRb was down-regulated, but pRb expression was unchanged in CTS-treated SiHa cells (Fig 4B). CTS dose-dependently increased the expression level of p53, resulting in modulation of downstream factors p21 and p27. As shown in Fig 4B, p21 and p27 expression levels were increased in a dose-dependent manner by CTS treatment as expected.

### CTS inhibits cell cycle progression and modulates cell cycle-related factors

**Fig 5:**
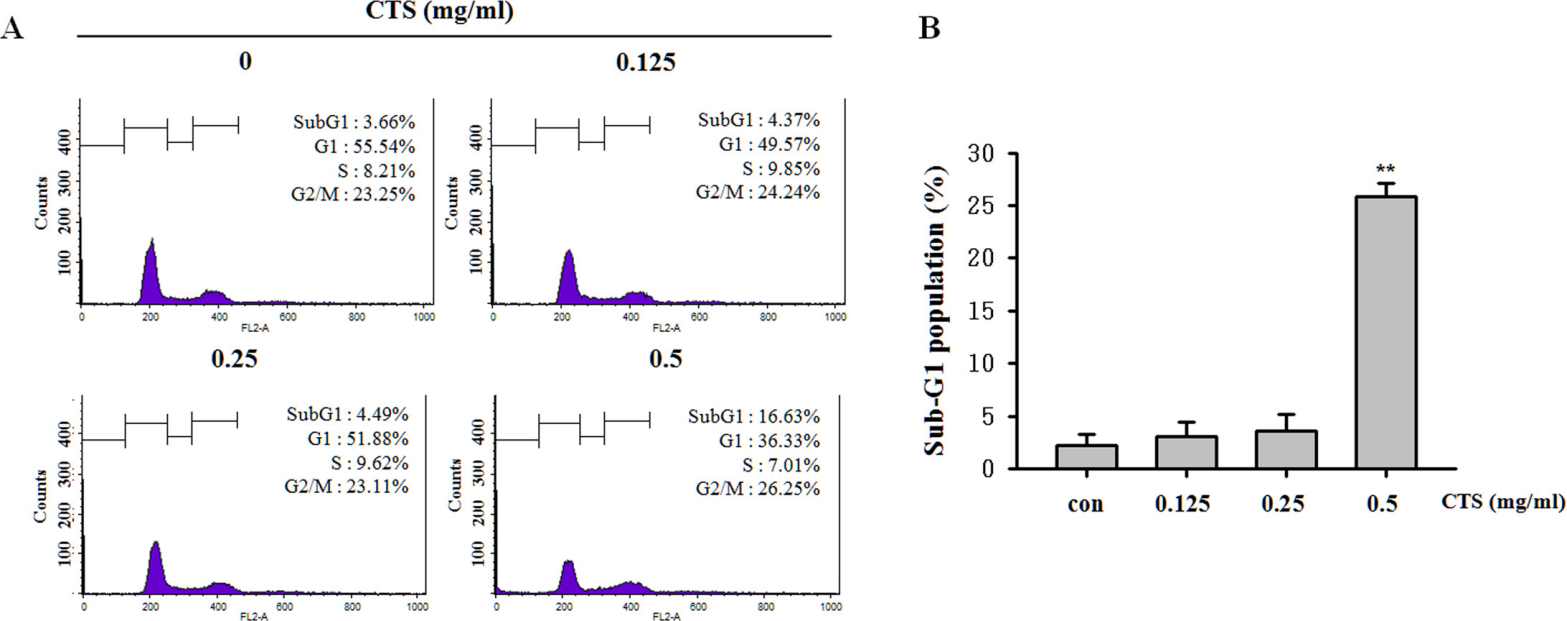
Effects of CTS on cell cycle status in SiHa cervical cancer cells. (A) Cell cycle profiles of CTS treated SiHa cells. (B) The proportion of cells in the sub-G1 phase. Results of **p < 0.005 were considered statistically significant. CTS-treated cells were compared to untreated cells.

To assess the effect of CTS on cell cycle progression, we examined cell cycle status by flow cytometry. In the previous experiments, expression levels of p53 and downstream genes p21 and p27 were increased following treatment with CTS (Fig 4B). Compared with the non-treated control cells, CTS-treated cells showed significant and dose-dependent accumulation in the sub-G1-phase (Fig 5B). However, there were no significant changes to the populations of cells in the G0/G1, S, and G2/M phases following treatment with CTS (Fig 5A).

### The effects of CTS are independent of the intrinsic pathway

Mitochondrial dysfunction is the most important factor in the intrinsic apoptosis pathway. When the mitochondria membrane potential collapses, cytochrome C is released into the cytosol, after which it forms the apoptosome with Apaf-1 and caspase-9 [21]. To measure mitochondrial membrane potential collapse following CTS treatment, we performed JC-1 staining and FACS analysis. As shown in S1A Fig, the JC-1 peak was not shifted following CTS treatment. In addition, the western blot analyses showed that CTS treatment did not alter the expression levels of pro-apoptotic factor Bax or those of anti-apoptotic factors Bcl-2 and Bcl-xL. In addition, cytochrome C was not released into the cytosol (S1B Fig). Thus, we conclude that amplification of the apoptotic signal after CTS treatment is independent of signaling via the intrinsic apoptosis pathway.

### CTS-induced apoptosis is mediated via death receptor signaling

**Fig 6:**
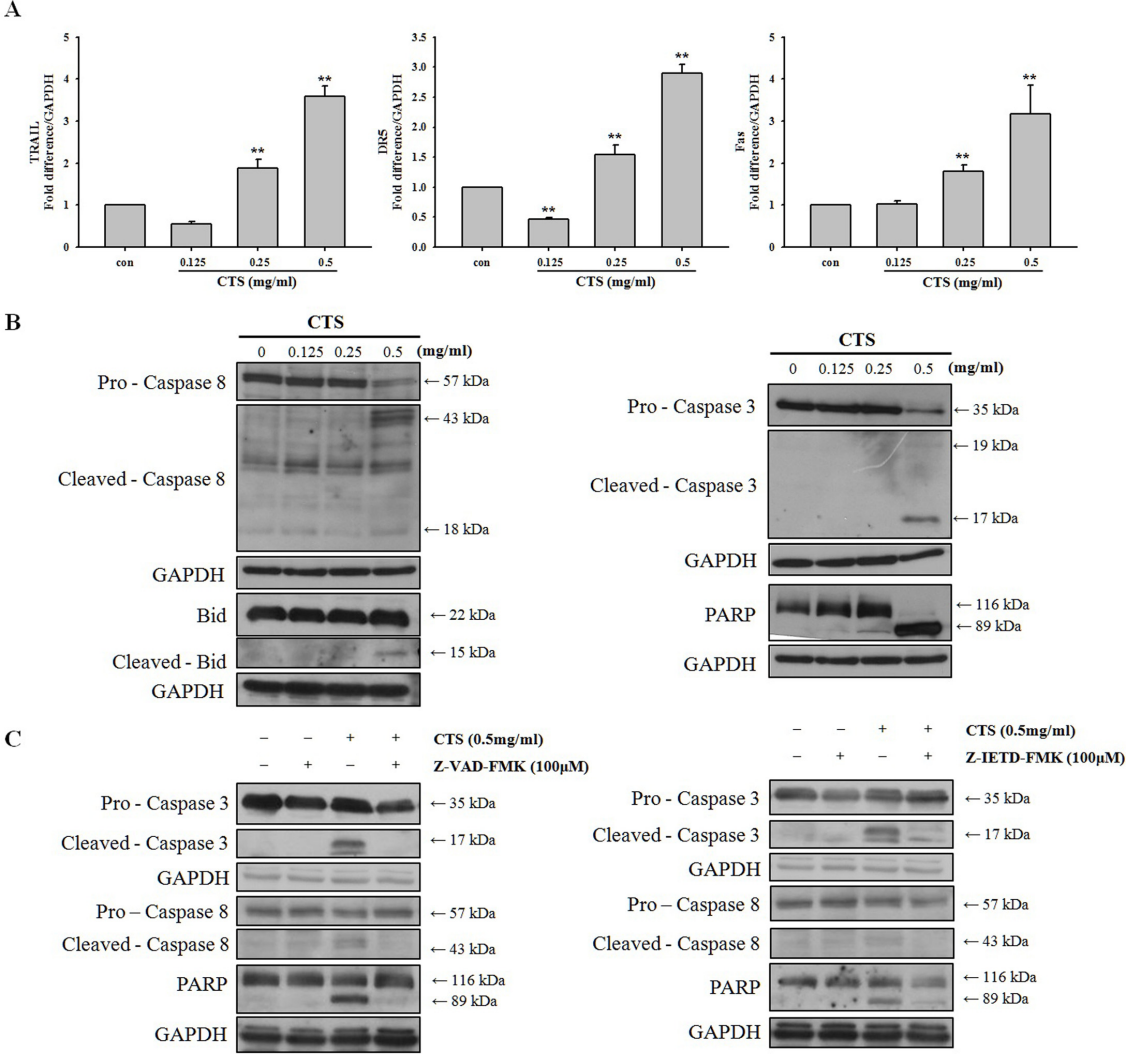
Effects of CTS on extrinsic pathway-related factors in SiHa cervical cancer cells. (A) mRNA levels of TRAIL, DR5 and Fas as detected by qRT-PCR. (B) Western blot analysis of extrinsic pathway-related factors. SiHa cells were treated with the indicated concentration of CTS for 24 h. (C) Western blot analysis of the effects of CTS following pretreatment with general caspase inhibitor Z-VAD-FMK or caspase-8 inhibitor Z-IETD-FMK in SiHa cells.

Because we demonstrated that the intrinsic apoptosis pathway was not involved in CTS-induced apoptosis, we next focused on the extrinsic apoptosis pathway. The extrinsic apoptosis pathway receives signals through the binding of extracellular death ligand proteins to proapoptotic death receptors (DRs) [22]. The extrinsic pathway transmits signals from extracellular ligands through proapoptotic DRs to the apoptotic caspase machinery [23]. In addition, Poly (ADP-ribose) polymerase (PARP) is involved in apoptosis, as well as a number of other cellular processes. We investigated expression levels of extrinsic pathway-related factors TRAIL, DR5, and Fas in SiHa cells following CTS treatment (Fig 6A). Our results showed that CTS treatment upregulates mRNA levels of TRAIL, DR5, and Fas. Protein expression levels of caspase-3, caspase-8, PARP, and Bid, were cleaved in a dose-dependent manner by CTS (Fig 6B). In addition, we identified the specific caspases involved in the pro-apoptotic mechanism of CTS. SiHa cells were pretreated with caspases inhibitors, including a general caspase inhibitor and a caspase-8 inhibitor. As shown in Fig 6C, pretreatment with general caspase inhibitor Z-VAD-FMK prior to CTS treatment significantly blocked CTS-induced apoptosis. A similar inhibitory effect on CTS-induced apoptosis was produced by caspase-8 inhibitor Z-IETD-FMK. These results show that caspase-3, caspase-8, and PARP are activated via DR-mediated signaling during CTS-induced apoptosis.

### Down-regulation of E6 and E7 genes enhanced CTS induced apoptosis

**Fig 7:**
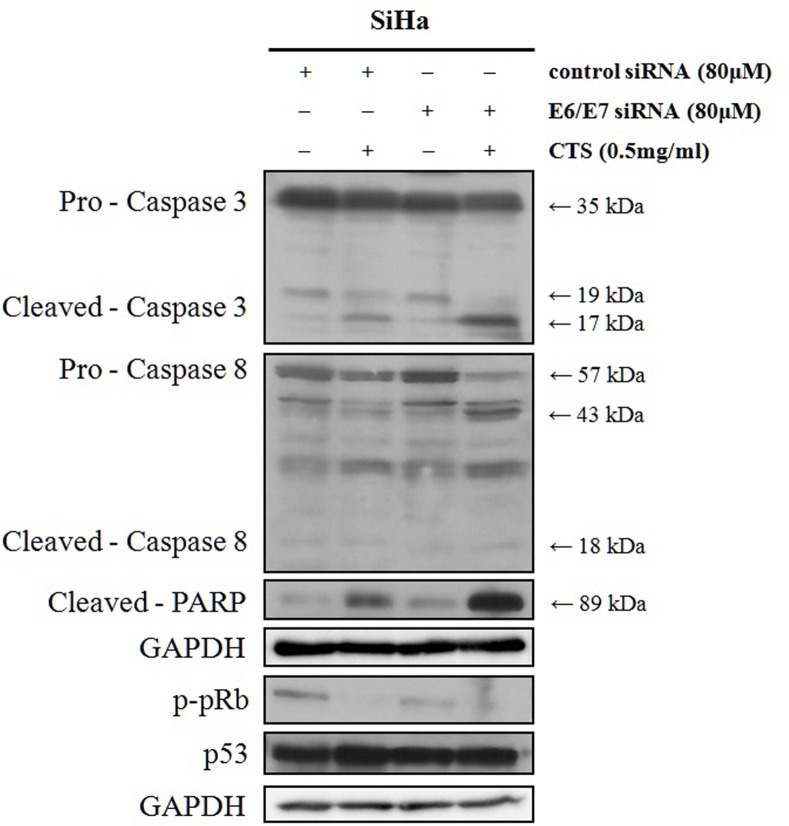
Effect of CTS and E6/E7 siRNAs in SiHa cervical cancer cells. Western blot analysis of apoptosis-related factors after transfection of control siRNA or E6/E7 targeting siRNA.

To investigate whether CTS-induced apoptosis would be affected by E6/E7 levels, E6/E7 siRNA was transfected and treated with CTS. Apoptosis activating proteins such as caspase-3, caspase-8, and PARP were more cleaved under E6/E7 siRNA transfection and CTS treatment (Fig 7). p-pRb expression level was decreased in E6/E7 siRNA transfected SiHa cells compared to control siRNA transfected cells. However, p53 expression level was not altered (Fig 7). These results show that CTS might support induction of apoptosis through down-regulation of E6/ E7 mRNA expressions.

## Discussion

The main objective of our study was to confirm the anti-cancer efficacy and associated mechanisms of CTS in human cervical cancer cells. CTS extract inhibited cervical cancer cell proliferation in HPV-positive SiHa and CaSki cells. The cytotoxic efficacy of CTS in HPV-16-positive SiHa cells was slightly better than its efficacy in CaSki cells (Fig 2). This effect might be due to the fact that the number of HPV genome copies in CaSki cells is higher than that of SiHa cells [24]. This finding suggests that the total number of HPV copies in the cells may have influenced their susceptibility to the proapoptotic effects of CTS [24]. In addition, we demonstrated that CTS down-regulated expression levels of oncogenes E6 and E7 in HPV-16-positive cell lines. As expected based on the decreased expression level of E6, we confirmed that p53 expression was increased in dose-dependent manner. Interestingly, decreased expression of E7 was not associated with altered pRb expression; however, p-pRb was decreased in a dose-dependent manner by CTS (Fig 4B). This result reminded earlier reports demonstrating that inhibition of E7 resulted in reduced phosphorylation of pRb without changing overall pRb protein expression [20].

CTS extract contains several phenolic compounds (Fig 1). Chlorogenic acid is present at the highest concentration among them. Chlorogenic acid has been reported to have anticancer, antioxidant, and antidiabetic effects [14, 25–26]. In addition, chlorogenic acid is the second major bioactive component in coffee after caffeine [25]. Chlorogenic acid stimulates glucose transport in L6 skeletal muscle via AMPK activation, which contributes to the beneficial effects for diabetes [25]. Moreover, chlorogenic acid is an antioxidant that may slow the release of glucose into the bloodstream after a meal [26]. In addition, chlorogenic acid can induce cellular DNA damage and apoptosis in lung cancer cells without affecting normal lung fibroblasts [14]. However, the anti-cancer effects of chlorogenic acid in cervical cancer cells have not been comprehensively investigated. Caffeic acids are found in many natural plants [27] and have been shown to suppress tumor growth by inhibiting tumor cell proliferation and enhancing antioxidant activity [28]. A previous study showed that caffeic acids inhibited proliferation, adhesion, and migration by A549 human lung cancer cells and HT29-D4 colon cancer cells [29]. Additionally, hesperidin, naringenin and quercetin have been reported to exhibit anti-cancer effects in several cancer cell types [16–17, 30–31].

Apoptosis is mediated by the intrinsic and extrinsic pathways. The intrinsic pathway is mediated by mitochondrial outer membrane permeabilization (MOMP) and cytochrome c release from the mitochondria into the cytoplasm [32–33]. In the cytosol, cytochrome c induces assembly of the apoptosome, which contains the adaptor protein Apaf-1 and apoptosis-initiating protease caspase-9. Apoptosome formation activates caspase-9, which activates effector caspases [34]. The extrinsic pathway receives signals through the binding of extracellular protein ligands to proapoptotic DRs located on the cell surface [23]. Although several DRs have been described, we focused on Fas (CD95) and DR5 and their respective ligands such as Fas ligand (Fas L) and TRAIL [35]. DRs have an intracellular death domain that recruits adapter proteins and caspase-8 [36]. Binding of the death ligand to the DR results in formation of the death-inducing signaling complex (DISC), composed of the death receptor, FADD and caspase-8 [37]. The DISC activates a downstream signaling cascade resulting in apoptosis [38–40]. We investigated which pathways are involved in apoptosis induced by CTS in SiHa cervical cancer cells. Although intrinsic pathway-related factors were not affected by CTS treatment (S1 Fig), expression levels of extrinsic pathway-related factors such as Fas, DR5, TRAIL, and caspase-8, were affected by CTS treatment (Fig 6).

Our results show that CTS has a profound anti-cancer effect against cervical cancer cells. This is the first demonstration of the ability of CTS to inhibit expression of HPV-16 E6 and E7 oncogenes, and to induce apoptosis mediated by the extrinsic pathway in cervical cancer cells. However, further studies should identify the specific compounds in CTS responsible for its anticancer effects.

## Supporting Information

S1 Fig Effects of CTS on mitochondrial membrane potential and cytochrome C release in SiHa cervical cancer cells. (A) The difference in JC-1 colors was analyzed by flow cytometry. JC-1 aggregates (orange) are a feature of healthy cells, whereas JC-1 monomers (green) are a feature of apoptotic cells. (B) Western blot analysis of anti-apoptotic factors Bcl-2 and Bcl-xL, pro-apoptotic factor Bax and cytochrome C in SiHa cervical cancer cells. SiHa cells were treated with the indicated concentration of CTS for 24 h.
(TIF)

## Abbreviations

HPV: human papilloma virus
CTS: *Cudrania tricuspidata* stem
DR: death receptor
TRAIL: TNF-related apoptosis-inducing ligand
PARP: poly (ADP-ribose) polymerase
