# Supplementary figures and images for: Cudrania tricuspidata Stem Extract Induces Apoptosis via the Extrinsic Pathway in SiHa Cervical Cancer Cells
Source: PLoS One. 2016 Mar 9;11(3):e0150235. doi: 10.1371/journal.pone.0150235 (PMC4784787; doi:10.1371/journal.pone.0150235)

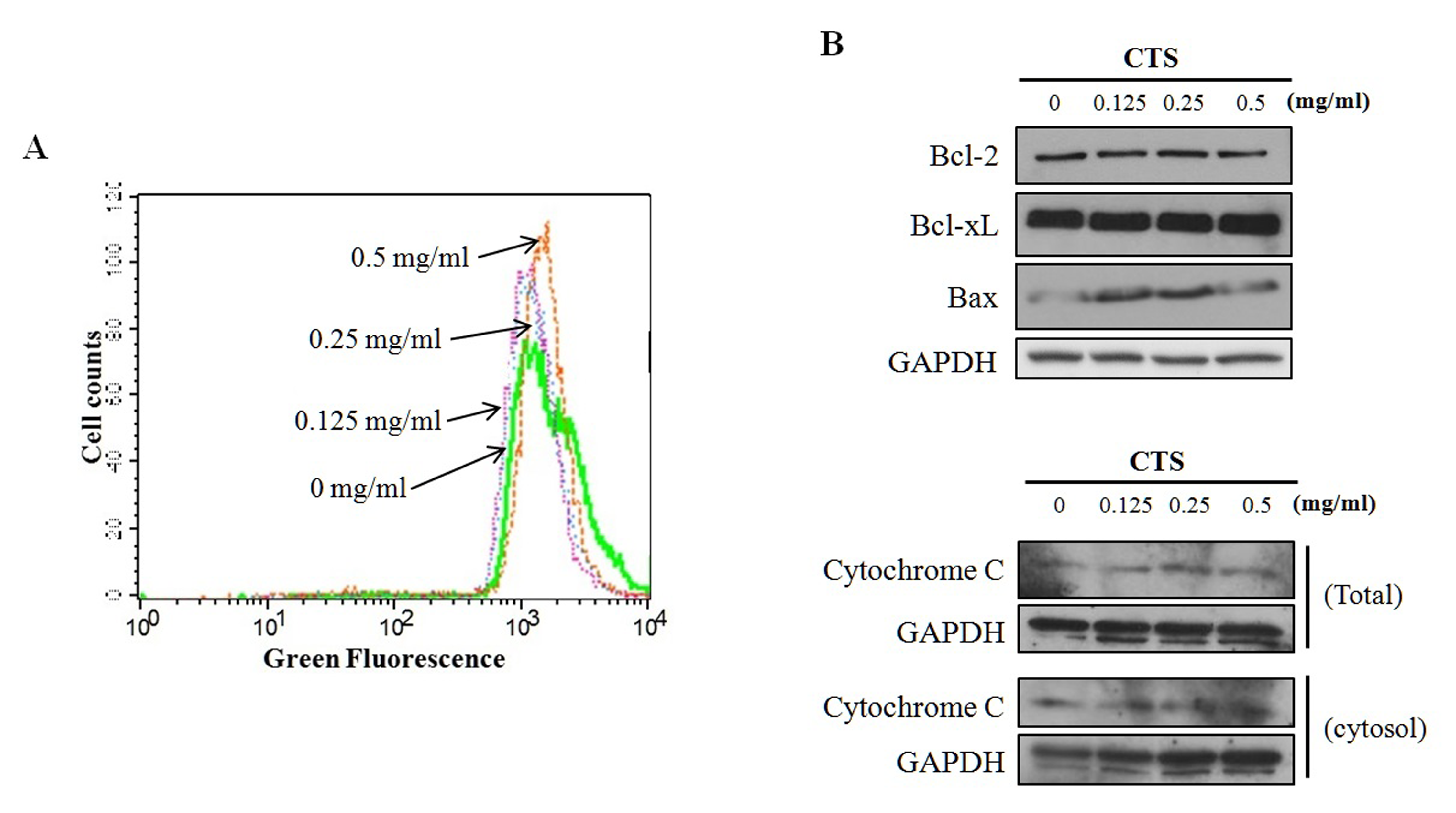

Supplement: S1 Fig — (A) The difference in JC-1 colors was analyzed by flow cytometry. JC-1 aggregates (orange) are a feature of healthy cells, whereas JC-1 monomers (green) are a feature of apoptotic cells. (B) Western blot analysis of anti-apoptotic factors Bcl-2 and Bcl-xL, pro-apoptotic factor Bax and cytochrome C in SiHa cervical cancer cells. SiHa cells were treated with the indicated concentration of CTS for 24 h. (TIF) [file pone.0150235.s001.tif]
